# *Account of the Progress of the Jennerian Inoculation upon the Continent, Contained in a Letter from* Dr. Friese, *of Breslaw, to* Mr. Ring, *of London*

**Published:** 1803-02-01

**Authors:** F. G. Friese


### *Account of the Progress of the Jennerian Inoculation upon the Continent, contained in a Letter from* Dr. Friese, *of Breslaw, to* Mr. King, *of London.*

Sir,

THE unremitting attention you have constantly paid to all the endeavours of the physicians of the Continent, to introduce and promote the new Jennerian inoculation in their respective countries, concerning which you have published from time to time many valuable articles in the Medical and Physical Journal, induces me to believe, that you will not take it amiss when I communicate to you a brief account of its introduction into this country.

From an assiduous perusal of some of the best English Medical Journals, I was early acquainted with the first publications of Drs. Jenner, Pearson, and Woodville. I soon furnished myself with the originals, and, in the beginning of the year 1800, wrote to Dr. Woodville for some matter. He very politely complied with my request; and sent me some dry vaccine virus, preserved between two square pieces of glass, at the same time giving me directions how to use it.

Before I received his answer, I was favoured with a letter by Dr. De Carro, who of his own accord, from an ardent desire of seeing Vaccination every where introduced, transmitted me a piece of impregnated linen, encouraging me by his own experience to make a trial. It was, however, some time, before I could find a parent who was willing to trust his children to the first experiment, particularly as the inoculation of the small-pox had been brought into great discredit with us, by the mode in which it had been performed.

After applying in vain to several of my medical brethren for co-operation, I was at length tired of those fruitless endeavours, when I had the good fortune to meet my friends Dr. Kruttge, Physician to the City Hospital, and Mr. Hartmann, Surgeon-major of a regiment of horse in our garrison. On the first of December 1800, we tried the matter received from Drs. De Carro and Woodville; but it proved effete. I then applied to the former gentleman, requesting him to send me a fresh supply.

In consequence of this application, on the 28th of December, I was enabled to inoculate three patients with Cow-pock matter, who took the disease in the most regular way; their pustules exactly corresponding with the prints affixed to Dr. Jenner's first publication,

After

After extending the practice to five other individuals with the same success, we thought it our duty to invite the public to avail themselves of the blessings of this important discovery, by a short advertisement in the newspapers. This was scarcely done, when the voice of a detractor resounded, with a view to frustrate all our attempts.

He had the malignant pleasure of seeing the progress of vaccine inoculation arrested for a short time; and it was owing to his bold and unwarrantable assertions, that the public mind was kept for some time in a state of suspense, and that vaccination was extinct near a month.

In the mean while Mr. Schwindt, surgeon-general to the Silesian troops, having been supplied with matter from Berlin, and joining us, we were fortunate enough to resuscitate the practice; and had the satisfaction to see three or four other physicians follow our example.

Thus a society for promoting Vaccine Inoculation was formed; and to this society the whole country is indebted for the propagation of the practice. I was charged by them to write a pamphlet on the subject, in order to inform the public of all the advantages to be derived from it. This was published gratis in the month of March, 1801; and it fully answered our expectations. The practice rapidly spread throughout Silesia; and the number of our patients daily increased.

At the end of June, I was commissioned by my colleagues to publish "A Continuation of Accounts concerning the Progress of Vaccination in Silesia," which contains important evidence in support of the preservative power of the new method, and a list of all the persons who had undergone Vaccine Inoculation.

The number of persons vaccinated by the associated physicians amounted at that time to 522, beside several hundred inoculated by other practitioners of the town, and a much greater number by physicians and surgeons in the country.

The new Inoculation thus becoming general, some blunders might well be expected. We therefore thought it incumbent on ourselves to publish some further remarks, containing rules for discriminating the genuine vaccine pustule from every spurious kind, together with directions how to perform the operation in the best manner hitherto known; and I am happy to assure you, that by these means many mischiefs have been prevented, which in other parts of Germany have so greatly retarded the adoption of this fortunate discovery.

In order to put the preservative virtue of the vaccine virus to the test of experiment, and to place it beyond all reasonable doubt, the society subjected twenty-six children, who had been previously vaccinated, to a public inoculation with fresh variolous matter; which they all resisted.

Such was the progress which vaccination had made, when the anniversary of our first trial occurred. We therefore deemed it a favorable opportunity to look with satisfaction on the result of our labours; and to celebrate this happy day. A select number of the friends of humanity were invited by the society to an entertainment. The print of the immortal Jenner was placed in view; suitable toasts were given, and an ode composed by our celebrated Prof. Fulleborn was sung, which gained the applause of every competent judge. An allegorical transparent picture, and another ode adapted to the subject, closed the joyful scene; the memory of which will never be erased from our minds. Life affords but few occasions for joy and conviviality like this.

The number of vaccinations in Breslaw now amounts to 1800, and that in the country to nearly 10,000. I have been so fortunate as to propagate Vaccine Inoculation to Moscow; where it commenced in the beginning of October, 1801, under the direction of Mr. Lindstrohm, Counsellor of State, and one of the Surgeons in ordinary to his Majesty the Emperor. The young Vaccinoff, of whom you have spoken, in the 38th Number of the Medical and Physical Journal, as the first child vaccinated in Russia, received the infection from an impregnated thread sent by me to Moscow.

In consequence of this success, Her Majesty, the Empress Dowager, has graciously presented me with a valuable brilliant ring, accompanied with a very polite letter.

My friend Mr. Lindstrohm, who is now at Vienna, attending the Grand Duke Constantine, informs me, by a letted dated the 30th of last month, that vaccination is now spread throughout the vast dominions of Russia.

I lately inoculated an ass, five months old, with vaccine matter. It was inserted into the teat; where it produced a small pustule, but no efflorescence.

Should this Report of the State of Vaccination in Silesia, be deemed worthy of insertion in the Medical and Physical Journal, I shall be obliged to you if you will transmit it to the Editors. I have the honor to be, Sir, with great respect,

Your obedient humble servant,

